# Uric Acid as an Etiological Factor*Read before the Alumni Association of the Southern Medical College, July 22, 1898, Atlanta, Ga.

**Published:** 1898-09

**Authors:** E. Van Goidtsnoven

**Affiliations:** Atlanta, Ga.; 102½ Whitehall street


### URIC ACID AS AN ETIOLOGICAL FACTOR.*

BY E. VAN GOIDTSNOVEN, A.M., M.D., ATLANTA, GA.

I doubt whether in the catalogue of etiological factors one can be found more common, more insidious in its evil doing, more metastatic in its manifestations, more hydra-headed in its stubborn resistance to treatment, more lethal in its results, and yet more ignored by the medical profession than uric acid.

Every stage of life, every condition of existence is susceptible of lithæmia. We can scarcely conceive of a disorder which, having baffled all attempts at research and treatment, is not suggestive or evocative of this pathological causation. The infant, the adolescent, the adult, the senile, the rich, the poor, the landlord, the peasant, the banker, the mechanic, the profession, the laity, all are subject to imperfect elimination, deficient excretion and consequent diathetic or cachectic lithæmia.

Urea is, so to speak, the last hystolitic product, the *ultima thule* of tissue decay which, incapable of being more highly organized, can no longer serve the purposes of life, and is therefore secreted by the urine.

Uric acid has many congeneric associates, such as Xanthine, Hypoxanthine, Creatine, Creatinine, Tyrosine, etc. They present many points of resemblance. Their chemical formulæ being almost identical, their relationship is closer than that of an isomeric nature. All are early excreta of tissue decay, constitute an array of powerful neurotic poisons, and when not removed from the organism through the instrumentality of katabolic agencies and the media of excretory organs, produce serious disorders, often ending in coma, convulsion and death.

It is a well-known fact that the formation of uric acid is antecedent to that of urea; that it occupies an intermediate position between the proteid compounds as they enter the system as nutrients and their final elimination into urea.

An excess of uric acid produces toxic phenomena, perversion of function and organic disorders throughout the whole economy.

To those who recognize a clinical and pathological relation between lithæmia and rheumatism, gout, eczema, asthma, dyspepsia, diabetes, Bright's disease, etc., etc., *it is somewhat amazing that the majority of the medical professsion, and especially the insurance examiners, should restrict the importance of urine analysis to diabetic and albuminous excreta.*

We all admit that nitrogenous waste creates a condition in the organism incompatible with health, and that the blood loaded therewith becomes the seat of chemical changes which

*Read before the Alumni Association of the Southern Medical College, July 22, 1898, Atlanta, Ga.

materially differ from those ordinarily taking place in its normal constituents. The particular chemical compounds formed under these circumstances are not readily excreted. Remaining in the blood, they accumulate, and minute bioplasts or leucocytes grow and multiply. At length an influence upon the nerves is exerted, and there ensue toxic phenomena, toxic manifestations of diseases which are auto-genetic, auto-infectious and frequently septic. Impaired vision, neuralgia, hypochondriasm, gout, rheumatism, eczema, dyspepsia, bronchorrhœa, bronchitis, colds, catarrhs of mucous membranes, diarrhea, diabetes mellitus and insipidus, many forms of gout and of chronic lithiasis, otherwise called latent and suppressed gout, such as valvulitis, prostatitis and even testitis. All these disorders can in many instances be traced to the presence of toxic by-products in the organism and especially of uric acid.

It is generally believed that nitrogenized waste passes off by the kidneys only. We should remember, however, that there is such a thing as metastasis of excretion; that the skin, the lungs, the bowels are emunctories of no less importance than the renal organs; that there exists between these excretory organs a complementary relation, and that if the function of one be interfered with, another performs it, to a certain extent. These are not diseases *per se.* They simply indicate that one organ is called upon to perform the duties of another organ temporarily affected with inadequacy or insufficiency. But in truly pathological conditions these compensatory actions and vicarious functions are increased to a burthensome degree and lead to gross disorders. Hence, we readily understand such outcome as above enumerated when uric acid, which is insoluble in the fluids of the body, is allowed to cumulate and remain within the economy, especially if there be hepatic or renal inadequacy.

I now come to a question which I consider of practical interest. How are we to detect the presence of uric acid and its toxic associates in the animal kingdom?

Whenever I am called to treat a patient complaining of any of the disorders above mentioned, if previously intractable, I prescribe terebene in fifteen-drop doses, to be triturated in sugar and administered every four hours. When the patient has taken from one to two drachms of terebene, I examine the urine, and the murexide test almost invariably *tells the tale.*

According to Dujardin-Beaumetz, terebinthinates introduced into the system, whether by the skin, lungs or stomach, enter the general circulation. They then decompose, and while the volatile substances are eliminated by the lungs, the fixed parts are excreted by the urine and exercise their individual properties upon the urinary passages, *provided the latter be in a permeable state.*

In health, and even in many a morbid condition, terebinthinates impart a characteristic odor to the urine—that of violets. In nephritric patients, however, these substances, when administered, gradually fail, with the progress of disease, to give rise to this odor. De Beauvais calls attention to the want of elimination of this odor in the last stage of Bright's disease.

Terebene is the terebinthinate *par excellence*. I use it:

First, as the best eliminator at my command, and, therefore, I value it as a therapeutic agent of the highest order.

Secondly, as an infallible indicator and enunciator of lithemic condition.

Thirdly, as a guide whose assistance is of considerable value in studying, ascertaining, and appreciating the successive stages of Bright's disease.

For more than ten years I have given this subject considerable thought, study and practical application. I now submit these views and suggestions to the medical profession in the hope that they will elicit consideration and no little criticism. If there be any chaff therein, let it be blown off. I shall be but too happy if only a few grains remain in the winnowing van *pro bono publico*.

How are we to treat these morbid conditions? It must be inferred from the above *expose* that the digestive organs constitute the *nidus* of all these troubles, and that the liver is the *fors et origo* of disease and calls on the vicarious functions of the kidneys, of the lungs and of the skin to come to its relief. Too much burthen is thus thrown upon the liver, whose function is the secretion of bile, the manufacture and storage of sugar and the destruction of worn-out blood cells. When too long overtaxed by indigestion, it can neither dehydrate the material which courses through it nor maintain its glycogenic functions, nor ooze out the amount of bile necessary to secure the aseptic condition of the intestines, nor throw off those refuse matters that are discharged in it. Hence auto-infection, sepsis and all its dreadful concomitants. What do these conditions mean?

There is nitrogenized waste in the blood.

The physician's duty here is manifold.

First.—He must cut down the amount of albuminoids eaten or drank, in order to reduce the demand upon the liver. The diet should be restricted to warm fluids, such as milk alone or with arrowroot, gruel, beef tea. Milk and Apollinaris water constitute an excellent diet. Meat should be stricken out entirely from the dietary until the patient has made considerable improvement. Outdoor exercise should be enforced.

Secondly.—He must sweep away the waste from the blood by a cathartic and alterative, preferably at bed-time.

Thirdly.—He must reduce the uric acid into urea and carbonic acid and so get rid of it by flushing the circulation.

The hepatic remedy which I prescribe under the name of "Fel. Bovis compound" contains blue-mass as a cathartic and alterative, ox-gall to promote the flow of bile and asepticize the intestines, ipecacuanha to increase the vascularity of the stomach and promote digestion, colocynth compound as an hydragogue and adjuvant to the blue-mass. To antagonize the fermentative and putrefactive changes that take place within the stomach; to eliminate the uric acid and its congeneric associates which poison the circulation, I flush the latter with a solution of sulphite of soda and salicylic acid in water of wintergreen, which I have named "Liquor Sulpho Salicylata." I use the above-named preparations in every case of lithæmia I am called upon to treat, and am yet to meet with *the first failure.*

Pepsin and its manifold preparations are utterly worthless in lithemic conditions. Pepsin is an animal ferment capable of digesting meat out of the body if in the presence of warmth and an acid fluid. This is all the testimony of our senses enables us to affirm after witnessing artificial digestion in a test tube.

When we attempt, however, to submit the stomach to the same test by filling it with albuminous food and injecting pepsin, ingluvin *et id omne genus*, we often fail to obtain the same *chemical* results, and not seldom encounter *physiological* disturbances.

*Comparisons are truly odious.* Nature never experiments. The physicist constantly does, and often presumes to speak "ex cathedra." Nature's laboratory is as infallible as self-asserting. The chemist's is incessantly tentative. Nature's digestive tube is acted upon by vital process and controlled by trophic centers. The chemist's digestive tube is an inert instrument, impermeable to sentient impressions, simply containing substances engaged in chemical but not physiological activities and obeying no law except that imparted to brute matter by chemical energy, under the eye of an investigating agent and the occasional stirring of his "pipette." One would infer from the way many gastric disturbances are treated nowadays that the majority of the medical profession have come to the conclusion that *dyspepsia* has evolutionized into *apepsia*, and that the gastric juice, an acid secretion formed in the epithelial cells of the gastric tubules, and containing a ferment named pepsin, is a thing of the past. Hence they pour in the stomach pepsin and hydrochloric acid as a most eligible substitute. "Evidently," says Wood, "one of two things is certain, either the present practice is ridiculously absurd, or else pepsin acts upon the stomach in some way as a stimulant."

All I can say in favor of pepsin is that it is a stimulant. If, to use the language of Fothergill, the activity of an organ is in direct relation to its blood supply, I know of no better or more rational plan to increase the vascularity of the stomach, and so improve digestion, than by the administration of such agents as arsenica, ipecacuanha, alcohol or any stomachic, together with antifermentative preparations, such as sulphite of soda or salicylic as above described.

Such a course has at least the merit of discarding empiricism and of being in accordance with common sense, which teaches us that in lithemic conditions an additional ferment is but an increased incubus to an already fermentative and putrefactive digestion, and is more than likely to aggravate the existing morbific process.

102½ Whitehall street.

---

### FRACTURE OF THE PENIS.

A man in a very drunken state had a powerful erection, and without any cause or reason known to himself he with both hands bent it forcibly backwards. He was conscious of something giving away, and awakened with the pain. The erection subsided after a time, and the pain also, buthe was very much surprised in the morning by the enormous dimensions the organ had assumed , and its color. He therefore hastened to the hospital. With the exception of the glans, the organ was swollen and of a dark violet color. On the dorsum was a painful thickening, when the surgeon suspected a rupture of the corpus cavenosum had taken place. The treatment consisted of rest in bed, cold compresses and lead lotion, and in fourteen days the normal color had returned, although the thickening remained almost unchanged. In six weeks the patient showed that, although a slight thichening still remained, no lasting injury had been sustained.—*Deutsche Medical Zeitung*, October 3, 1896.

---

### ON THE HEALING OF WOUNDS IN THE NEGRO RACES.

Plehn, in the *Deut. med. Woch.*, 34, 1896, as the result of his studies, finds that infected wounds are rare among the negro races in Africa, and, moreover, severe wounds seem to heal sooner than do like wounds occuring in Europeans. He also concludes that the specific pus organisms are not common in Kamerun, and that the tissues of the negro are distinctly resistant.

